# “Impact of COVID-19 pandemic lockdown on early onset of puberty: experience of an Italian tertiary center”

**DOI:** 10.1186/s13052-021-01015-6

**Published:** 2021-03-05

**Authors:** Martina Verzani, Carla Bizzarri, Laura Chioma, Giorgia Bottaro, Stefania Pedicelli, Marco Cappa

**Affiliations:** Unit of Endocrinology, Bambino Gesù Children’s Hospital, IRCCS, Piazza Sant’Onofrio 4, 00165 Rome, Italy

**Keywords:** Precocious puberty, SARS-CoV2 pandemic, COVID-19 lockdown, Italy

## Abstract

At the end of 2019, an emerging atypical pneumonia called COVID-19 (coronavirus disease 2019), caused by the novel coronavirus defined as SARS-CoV-2 (Severe Acute Respiratory Syndrome Coronavirus 2), was first reported. COVID-19 rapidly expanded leading to an epidemic in China, followed by a global pandemic during the year 2020. In few weeks Italy was assaulted by a severe health emergency, constraining the Italian government to put in place extraordinary restrictive measures, such as school closures and a strict lockdown extended to the entire country at the beginning of March 2020. Since the beginning of lockdown, the Endocrinology Unit of Bambino Gesù Children’s Hospital has recorded a rapid increase of the outpatient consultations for suspected precocious or early puberty. We have now retrospectively analyzed all the consultations recorded in the database of our outpatient clinic from March to September 2020, and compared them with the consultations recorded in the same database from March to September 2019. Our preliminary data suggest a significant increase of precocious puberty cases in girls during the first period of COVID-19 pandemic. Further investigations in larger cohorts of children are needed in order to correlate the observed increase of precocious puberty with specific pathogenic factors.

## Main text

At the end of 2019, the novel SARS-CoV-2 coronavirus (Severe Acute Respiratory Syndrome Coronavirus 2) was identified as the cause of a cluster of atypical pneumonia cases in Wuhan, a city in the Hubei Province, China [1, 2]. This emerging disease called COVID-19 (coronavirus disease 2019) rapidly expanded, leading to an epidemic in China, followed by a global pandemic [3].

In February 2020, two main COVID-19 clusters were identified in northern Italy and several municipalities were placed under quarantine. Due to the high transmission rate and the severity of the clinical picture, a sharp increase of the emergency admissions was observed, with intensive care units saturation. In order to counteract this severe health emergency, at the beginning of March 2020 the Italian government put in place extraordinary restrictive measures, such as school closures and a strict lockdown extended to the entire country [4]. During the first phase, Italian citizens were required to stay at home with the introduction of the so-called “smart-working”. Education also changed dramatically, with the distinctive rise of e-learning, whereby teaching was undertaken remotely and on digital platforms and distance learning until the end of the school year in June 2020. Moreover, any kind of both outdoor and indoor physical activity was prohibited, except home activities.

During the lockdown, we observed an evident increase in the number of outpatient consultations for suspected precocious or early puberty.

In order to clarify this preliminary observation, we have retrospectively analyzed all the consultations recorded in the outpatient clinic database of the Endocrinology Unit of Bambino Gesù Children’s Hospital, from the beginning of lockdown in March 2020 to September 2020, in comparison with the consultations recorded in the database in the same period of 2019.

In 2020, 246 patients were referred for suspected precocious puberty, compared with 118 consultations recorded in 2019 (+ 108%). Consultations for premature thelarche in girls younger than 3 years (25 girls in 2019 and 22 girls in 2020) were excluded. Our preliminary data showed a prevalent increase of female cases (215 subjects in 2020 versus 87 patients in 2019), whereas no difference was observed in male patients (9 in 2020 versus 6 in 2019). Most patients were Caucasian of European ancestry in both observation years (217/224 in 2020 and 90/93 in 2019). Clinical regression of thelarche was described at the second observation in 15.1% of cases in 2019 and 17% of cases in 2020. Clinical characteristics of the two groups, as age, height, body weight and body mass index (BMI) with relative gender- and age- specific standard deviation scores (SDS), are reported in Table 1. Data are expressed as mean ± SD, if not differently indicated. No significant differences of the anthropometric parameters were observed between girls with precocious puberty observed in 2019 and 2020.

**Table 1:** Anthropometric parameters of 2019 and 2020 patients referred for precocious puberty

	2019			2020			***p***
	Female	Male	Total	Female	Male	Total	
**Number**	87	6	93	215	9	224	0.38*
**Age (year)**	7.51 ± 1.07	7.97 ± 2.80	7.54 ± 1.23	7.33 ± 0.86	8.14 ± 1.12	7.37 ± 8.79	0.151
**Height (cm)**	129.06 ± 9.2	128 ± 18.08	128.99 ± 9.84	126.96 ± 7.41	135 ± 11.28	127.28 ± 7.73	0.100
**Height SDS**	0.81 ± 1.11	0.35 ± 1.28	0.78 ± 1.12	0.63 ± 1.09	1.14 ± 1.33	0.65 ± 1.10	0.375
**Weight (kg)**	30.67 ± 7.77	28.97 ± 10.70	30.56 ± 7.93	28.27 ± 6.23	40.21 ± 15.38	28.75 ± 7.16	0.048
**Weight SDS**	1.05 ± 1.13	0.43 ± 1.69	1.01 ± 1.17	0.8 ± 1.07	1.66 ± 1.14	0.84 ± 1.09	0.211
**BMI (kg/m**^**2**^**)**	18.19 ± 3.02	17.35 ± 3.32	18.13 ± 3.03	17.39 ± 2.58	21.43 ± 5.63	17.55 ± 2.86	0.105
**BMI SDS**	1.24 ± 1.45	0.14 ± 1.81	1.18 ± 1.48	0.90 ± 1.34	2.09 ± 1.36	0.95 ± 1.36	0.186

Legend: Anthropometric parameters are expressed as mean ± SD if not differently indicated. Independent Student’s t-test was used to compare means between 2019 and 2020 groups. Statistical differences between prevalences were assessed by Fisher’s exact test. *Difference in sex prevalence between 2019 and 2020 groups.

Our preliminary data suggest a significant increase in cases of precocious puberty in girls from the beginning of COVID-19 pandemic, compared to the same 6-months period of 2019. A recent study reported an increased incidence of newly diagnosed cases of central precocious puberty  and a faster pubertal progression during and after lockdown [5]. Further studies, involving several Italian pediatric centers are needed to confirm this phenomenon on a larger scale, and to correlate this increase with specific pathogenetic factors.

During the first phase of the Italian lockdown, evident changes in everyday life were strictly imposed, as school closures and stop of sport activity. Families were forced to stay at home, except for emergency reasons, with more opportunities for hypercaloric food consumption and overnutrition. Education changes with a significant rise of e-learning, extremely uncommon in primary schools before the pandemic, resulted in a larger use of electronic devices, as tablets or personal computers, among children. Finally, it has recently been observed that stress-related symptoms due to the serious national health emergency have become more common in both adults and children [6].

In conclusion, our preliminary data suggest a significant increase of precocious puberty cases in girls during the lockdown due to COVID-19 pandemic. Further investigations are required in order to correlate the increase of precocious puberty observed in girls to specific pathogenetic factors. An Italian multicentric study protocol involving other Italian pediatric centers is under development and will be proposed in the near future.

## Data Availability

The datasets used and/or analyzed during the current study are available from the corresponding author on reasonable request.

## Abbreviations

SARS-CoV-2: Severe acute respiratory syndrome coronavirus 2
COVID-19: Coronavirus disease 2019
SD: Standard deviation
SDS: Standard deviations score
BMI: Body mass index
